# Integrated Approaches and Practical Recommendations in Patient Care Identified with 5q Spinal Muscular Atrophy through Newborn Screening

**DOI:** 10.3390/genes15070858

**Published:** 2024-06-29

**Authors:** Vanessa L. Romanelli Tavares, Rodrigo Holanda Mendonça, Maytê S. Toledo, Sônia M. Hadachi, Carmela M. Grindler, Edmar Zanoteli, Wilson Marques, Acary S. B. Oliveira, Paulo Breinis, Maria da P. A. Morita, Marcondes C. França

**Affiliations:** 1 Newborn Screening Reference Center, Instituto Jô Clemente (IJC), São Paulo 04040-033, Brazil; 2 Department of Neurology, Faculdade de Medicina, Universidade de São Paulo (FMUSP), São Paulo 05403-010, Brazil; 3 Secretaria de Estado da Saúde (Governo do Estado de São Paulo), São Paulo 01027-000, Brazil; 4 Hospital das Clínicas da Faculdade de Medicina da USP de Ribeirão Preto (HC/FMUSP-RP, São Paulo), Ribeirão Preto 14015-010, Brazil; 5 Motor Neuron Disease Unit, Division of Neuromuscular Diseases, Federal University of Sao Paulo (UNIFESP), Sao Paulo 04039-060, Brazil; 6 Faculdade de Medicina do ABC, Santo André 09060-870, Brazil; 7 Department of Pediatric Neurology, Irmandade da Santa Casa de Misericórdia de São Paulo, São Paulo 01221-010, Brazil; 8 Hospital de Base de São José do Rio Preto, São José do Rio Preto 15090-000, Brazil; 9 Department of Neurology, Universidade Estadual de Campinas (UNICAMP), Campinas 13083-888, Brazil

**Keywords:** *SMN1/SMN2*, genetic tests, modifying therapies, Spinal Muscular Atrophy, newborn screening, Latin America and Caribbean (LAC)

## Abstract

In recent years, significant progress has been made in 5q Spinal Muscular Atrophy therapeutics, emphasizing the importance of early diagnosis and intervention for better clinical outcomes. Characterized by spinal cord motor neuron degeneration, 5q-SMA leads to muscle weakness, swallowing difficulties, respiratory insufficiency, and skeletal deformities. Recognizing the pre-symptomatic phases supported by screening and confirmatory genetic tests is crucial for early diagnosis. This work addresses key considerations in implementing 5q-SMA screening within the Brazilian National Newborn Screening Program and explores Brazil’s unique challenges and opportunities, including genetic tests, time-to-patient referral to specialized centers, program follow-up, and treatment algorithms. We aim to guide healthcare professionals and policymakers, facilitating global discussions, including Latin American countries, and knowledge-sharing on this critical subject to improve the care for newborns identified with 5q SMA.

## 1. Introduction

Spinal Muscular Atrophy (SMA) refers to a group of hereditary neuromuscular diseases characterized by the degeneration of motor neurons in the spinal cord. The predominant form, 5q-SMA, is caused by homozygous deletion in the Survival Motor Neuron 1 (*SMN1*) gene in 95% of the cases. The remaining cases are caused by compound heterozygous variants or, in very rare ones, by subtle variants in homozygosis (small insertions/deletions and point variants) [1,2]. 5q-SMA presented with a broad spectrum of clinical manifestations mainly related to the number of copies of a paralog gene, the Survival Motor Neuron 2 (*SMN2*), which produces around 10% of functional Survival Motor Neuron (SMN) protein [3]. In all cases, the disease is characterized by proximal muscle weakness, and the natural course of the most severe forms also present with respiratory muscle weakness and progressive bulbar involvement, resulting in ventilatory insufficiency and contributing to dysphagia, respectively [4,5,6]. 5q-SMA worldwide incidence varies according to studies and analyzed cohorts: it has been estimated in about 1 in 12,000 live births [7] considering symptomatic patients and 1:14,848 on average, considering NBS programs in Germany, USA, Belgium, Japan, and Australia [8].

The landscape of 5q-SMA has entered a new era with the development of modifier genetic therapies since 2016, with the first 5q-SMA drug approval by the Food and Drug Administration (FDA, USA): Nusinersen. Following that, Nusinersen and two other drugs were approved (Risdiplam and Onasemnogene abeparvovec) not only by the FDA but also by other drug regulators such as the EMA (European Medicines Agency, Europe) and Agência Nacional de Vigilância Sanitária (ANVISA, Brazil). A recent systematic review regarding treatment outcomes in 5q-SMA patients, identified by newborn screening, reported that more than 90% of cases with >3 *SMN2* copies achieved normal development. Moreover, studies have demonstrated that even patients with 2 *SMN2* copies develop better outcomes when treated pre-symptomatically compared to untreated patients [8]. The impact of therapy advances goes beyond the clinical domain, also shaping new expectations for patients, caregivers, and families.

Consequently, a profound understanding of the prenatal and pre-symptomatic postnatal phases of the disease has become essential, offering opportunities for early diagnosis and prompt therapeutic intervention [9,10,11]. While several articles have reported on the local implementation of SMA newborn screening (NBS) and updates in their clinical practices [12,13,14,15,16,17,18,19], Latin America and Caribbean (LAC) remains underrepresented in the literature, with limited descriptions of the 5q-SMA population [20,21,22].

The attention of the LAC academic community toward publications addressing test validation, implementation costs, and a general overview of the adoption of NBS programs for rare genetic diseases has been increasing in recent years [21,22,23,24,25,26]. It is observed that NBS is subject to ongoing evaluations and continuous improvements. Moreover, it is important to emphasize that NBS is a public health program aimed at identifying newborns with treatable genetic conditions before they show symptoms, enabling the early initiation of treatment to prevent or minimize the effects of the disease. Additionally, it is noteworthy that NBS and its expansion to cover more disorders are just one part of a broader system of care for newborns with genetic disorders. Follow-up tests, diagnosis, and timely treatment are essential components for effective NBS implementation [23]. In this scenario, Brazil, as one of the LAC countries, alongside Argentina, plays an important role in creating approaches in health, developing clinical trials, and having qualified centers for the testing of neuromuscular disorders, especially concerning SMA [21,27].

Therefore, the aim of this work is to critically analyze opportunities and challenges associated with NBS in the LAC, with a primary focus on Brazil and the 5q-SMA. Specifically, it seeks to introduce current diagnosis and clinical approaches to 5q-SMA pre-symptomatic patients identified through NBS, supporting workflows as a guide for other newborn screening reference programs in their respective regions. To achieve this, a panel of key health professionals from São Paulo state, Brazil, was convened to develop an expert guide for diagnosis, follow-up, and treatment of 5q-SMA identified by NBS.

## 2. Methods

The Jô Clemente Institute (*Instituto Jô Clemente*, IJC), a reference service in newborn screening accredited by the Brazilian Ministry of Health, in collaboration with the Health Department of the state of São Paulo, invited specialists to compound an expert panel for 5q-SMA pre-symptomatic cases. The panel was composed of physicians specialized in genetics, neurology, and neuropediatric (n = 7), a motor physiotherapist specialized in neuromuscular diseases (n = 1), a geneticist (n = 1), and a pharmacist (n = 1) with a huge expertise in neonatal screening programs. The specialists were organized into working subgroups based on their expertise in areas including molecular diagnosis, genetic counseling, worldwide neonatal screening for 5q-SMA, patient referral and follow-up, and clinical treatment. Two meetings were conducted to present and discuss each topic and outline the guidelines to be included as a reference guide. Each subgroup structured a small document to be used as a guide for the final document. The literature review was performed using Embase and Medline through PubMed (including articles in English and Portuguese). The complete list of search terms can be found in Supplementary Material Table S1. All collaborators revised the final text.

## 3. Results

### 3.1. NBS in Latin America at a Glance

LACs are regions, territories, or countries belonging to the American continent whose geographical definition has derived from a complex historical-cultural aspect. Latin American countries from South America, Central America, Mexico, and some Caribbean locations (generally consisting of islands bathed by the Caribbean Sea) were initially grouped based on linguistic factors, as speakers of languages derived from Latin. Collectively, they form what we commonly refer to as the LAC. Another definition was based on “the confluence of profoundly disparate contingents in their racial, cultural, and linguistic characteristics, as a byproduct of European colonial projects” [28]. Several researchers present deeper issues with the aim of understanding and comprehending the conception of these regions and what they represent today.

The LAC has a population of approximately 669,416,054 million people distributed across 33 countries with marked political, cultural, social, economic, and healthcare system differences [29,30]. The introduction of NBS has experienced significant implementation time differences in each of their countries, probably related to a combination of unique economic, technical, logistical, social, cultural, and political challenges [24,25,31,32].

**Figure 1:**
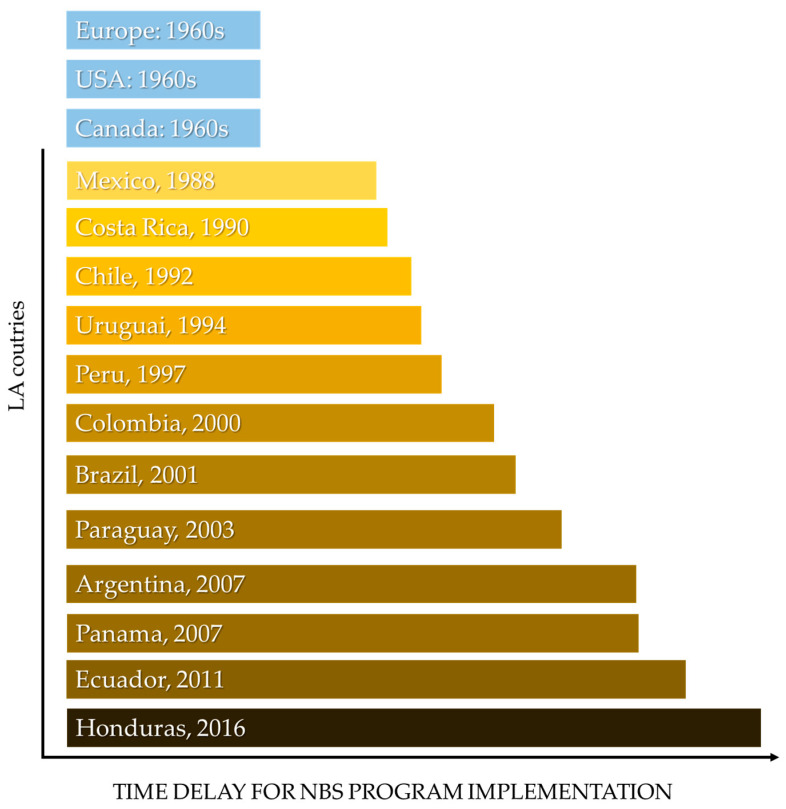
Time overview for newborn screening implementation in Latin American countries. Figure adapted from [23,24,32]. Mexico: The obligation to prevent mental retardation due to HC was established in 1988 following the publication of Technical Standard 321 in the Official Gazette of the Federation [34]. Costa Rica: In March 1990, the National Neonatal Screening Program was implemented through Executive Decree No. 19504-S, marking the commencement of neonatal screening in the country [35]. Chile: In 1992, Chile established the Neonatal Research Program [36]. Uruguay: Neonatal screening in Uruguay began in August 1990 at the Health Sector Maternity of the Banco de Previsión Social. Mandatory Neonatal Screening (PN) for congenital hypothyroidism was established in 1994, followed by the inclusion of other conditions in subsequent years [37]. Peru: In 1997, Peru adopted neonatal screening for congenital hypothyroidism, as outlined by Resolution 494-97-SA/DM from the Ministry of Health (MINSA) [38]. Colombia: neonatal screening for congenital hypothyroidism (HC) is not only addressed in Resolution 412 of 2000 but is also reinforced by various laws, decrees, resolutions, and rulings. These legal instruments underscore the obligatory nature and prompt implementation of expanded neonatal screening across the country, emphasizing it as a fundamental right for all newborns [39]. Brazil: In 2001, the Ministry of Health, through the Department of Health Assistance, undertook a reassessment of Neonatal Screening in the Unified Health System (SUS), leading to the publication of Ministerial Ordinance No. 822 on 6 June 2001, creating the National Neonatal Screening Program (in Portuguese: *Programa Nacional de Triagem Neonatal*—PNTN) [40]. Paraguay: In 2003, Paraguay created the National Neonatal Detection Program (PNDN) for the prevention of cystic fibrosis and mental retardation, making it mandatory to detect, diagnose, and treat these conditions in newborns [41]. Argentina: In 2007, Argentina strengthened the early detection of congenital diseases with the National Program for Strengthening the Early Detection of Congenital Diseases, established through the 2006 Decree, Resolution No. 1612/06, and Law 26.279/2007 [23]. Panama: Recognizing the importance of neonatal screening, Panama enacted Law 4 on 8 January 2007, establishing the mandatory Screening Program and dictating other provisions to ensure effective implementation [42]. Ecuador: The Neonatal Metabolic Screening in Ecuador commenced on 2 December 2011 [43]. Honduras: In September 2016, the National Congress of Honduras approved the “Mandatory Neonatal Screening Law”, resulting in the progressive implementation of a screening plan in SESAL and IHSS facilities [37].

The first assay implemented in NBS globally was for phenylketonuria (PKU), developed by Robert Guthrie in 1963. In the mid-1970s, the LAC witnessed the beginning of the first NBS programs in Mexico and Brazil [32]. Notably, PKU screening started in Brazil in 1976, followed by the screening of congenital hypothyroidism in 1980. However, it was only in 1990 that the screening of both diseases was regulated by federal law in the public health system, marking the initial step toward the National Program of NBS in Brazil, which materialized in 2001 [33]. Figure 1 illustrates a timeline of newborn screening programs performed in each Latin American country from a perspective of European, USA, and Canadian implementation (adapted from [23,24,32]).

In countries with incomplete or underdeveloped newborn screening programs, challenges can pass through legal and economic factors, resulting in inequitable coverage between public and private sectors and even a lack of connection between screening laboratories and specialized services, resulting in inadequate treatment coverage and decentralized follow-up without central coordination [24]. Despite the significant growth in NBS activities in the LAC over the last decade, another publication in 2021 emphasizes the persistence of major challenges, such as (1) the lack of national records hindering effective assessment, (2) political and economic crises threatening program continuity, and (3) technological disparity compared to developed countries (e.g., the delayed incorporation of mass spectrometry (MS/MS) in NBS) [32].

In summary, LAC countries must not only bridge the historical gap in NBS activities but also navigate their intricate economic, social, and political landscapes. Investing in and progressing toward acquiring and developing new technologies are essential steps to create an ideal scenario for sustainable and effective newborn screening programs across the region. This requires a concerted effort to address challenges, enhance coordination, and prioritize the allocation of resources for the betterment of neonatal health in the LAC.

#### Brazilian Neonatal Screening Program

Brazil is the largest country in the LAC, with approximately 8.5 million square kilometers in territorial area and a population of around 203 million people (IBGE census 2022, consulting in [44]). Currently, in Brazil, more than 80% of newborns are screened through Neonatal Screening Reference Services (in Portuguese: *Serviço de Referência de Triagem Neonatal*, SRTN), despite some regional differences being observed.

The National Neonatal Screening Program (in Portuguese: *Programa Nacional de Triagem Neonatal*—PNTN) started on 6 June 2001 and includes mandatory screening for six diseases: phenylketonuria (PKU), congenital hypothyroidism (CH), hemoglobinopathies, cystic fibrosis (CF), congenital adrenal hyperplasia (CAH) and biotinidase deficiency [45]. In 2021, a Brazilian federal law (number 14,154) was approved, establishing the obligation for all states to implement an expanded NBS panel by 2022—something that has not yet happened. The expansion is intended to implement the screening gradually, in five phases for (1) congenital toxoplasmosis (currently ongoing), (2) galactosemias, aminoacidopathies, urea cycle disorders, and disorders of fatty acid β-oxidation, (3) lysosomal diseases, (4) primary immunodeficiencies, and (5) 5q SMA, besides six diseases already screened in the current basic test. The number of NBS tests is estimated to increase from 6 to 53 treatable diseases [46].

Specifically, the inclusion of 5q-SMA in the expanded PNTN became possible with the approval of drugs that have dramatically changed the course of this neurodegenerative disease. This decision aligns with the criteria set by the World Health Organization (WHO) and the Brazilian Ministry of Health for diseases to be included in the NBS panel [47,48].

### 3.2. Global Implementation of Newborn Screening for 5q-SMA

The critical need for early intervention in patients affected by 5q-SMA, along with the proven benefits of pre-symptomatic treatment, has driven global efforts to implement neonatal screening programs. Among worldwide initiatives, there is a strong similarity in the design of pilot studies. In this approach, the diagnostic method is consolidated basically into two phases: initial screening (first-tier test) and confirmatory tests (second-tier test). Detected cases per number of screened newborns in 12 complete pilot studies are summarized and can be seen in Table 1.

Some important pilot studies that might help other countries implement 5q-SMA NBS include Belgium. The Belgian Pilot Study for 5q-SMA NBS was notable for showing that a pilot project can be rapidly transitioned into an official NBS program. Initially covering 17,000 newborns, its expansion reached about 55,000 births annually, and a total of 136,339 neonates were screened in the pilot. Nine cases of 5q-SMA were identified, and one case was reported after presenting symptoms with a compound heterozygous variant. The necessity of establishing a well-defined governance structure among the leaders of the pilot study (including the regional agency overseeing NBS and the NBS centers), along with the crucial involvement of key stakeholders (such as patient advocacy groups, neuromuscular reference centers, and engagement via social media by NBS centers), emerged as pivotal lessons learned during implementation. Taken together, these factors greatly facilitated a swift and seamless transition to an official program [13,49].

Another lesson learned came from the German Pilot Program, conducted between 2018 and 2021. The transition from the pilot project to a nationwide 5q-SMA NBS screening was reported. The authors emphasize the engagement of multiple stakeholders and cooperation between screening laboratories and local neuromuscular centers for *SMN2* determination and patient referral since the current NBS is the majority concerned with metabolic and endocrine centers. Moreover, a large discussion is made regarding the time required for treatment preparation while transitioning to a nationwide screening, such as sample transportation, insurance issues, and reimbursement for therapies [50].

Although these studies described a smooth transition from pilot to national implementation, the experience of Ontario, Canada, recommends continual assessment of the newborn screening program, incorporating the revision of existing algorithms as new evidence, drug reimbursement guidelines, and clinical consensuses come to light [15].

Regarding the methodologies used in the first- and second-tier tests, the Moscow Pilot Program stands out as the only one, thus far, to employ a melt assay (qPCR) in the screening test. Additionally, they conduct restriction fragment length polymorphism (PCR-RFLP) on dried blood samples (DBS) to confirm positive results. For further confirmation, a fresh blood sample is collected to perform Multiplex Ligation-dependent Probe Amplification (MLPA), assessing both *SMN1* and *SMN2* copies, with results available within 7 to 12 days of life (DoL) [19].

Last but not least, it is of utmost importance to also mention the United States’ experience with 5q-SMA implementation, included in the Recommended Uniform Screening Panel (RUSP). As of the latest update on 2 January 2024, 5q-SMA screening has been implemented in all 50 states plus Washington, D.C., covering 100% of newborns, as reported by Cure SMA [51]. Hale and collaborators [14] summarize the challenges of 5q-SMA NBS in the USA as follows: (1) deciding whether determining the number of copies of *SMN2* should be a state decision, (2) a retrospective systematic analysis will help to better understand the clinical progression in 5q-SMA cases with 4 copies of *SMN2*, and (3) acknowledging that insurance authorization for treatment can be lengthy, potentially delaying the start of recommended treatment.

**Figure 2:**
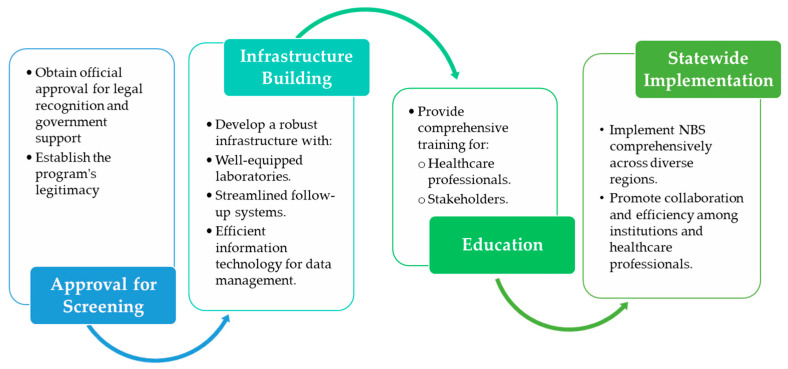
Overview of NBS phases of implementation. The final phase involves the comprehensive implementation of the NBS program across the entire state, based on [54].

Therefore, we conclude that several of these experiences and advancements should also apply to different governments and countries, and it is essential to discuss and incorporate them into the strategic planning of other countries in the process of implementing the 5q-SMA NBS. The four critical phases for the success of establishing a robust NBS program are described in Figure 2 [52].

**Table 1:** Number of identified patients with 5q-SMA in worldwide NBS Pilot studies.

Year	Country	Detected Cases per Number of Newborns	Reference
2018	Australia	18:202,388	[53,54]
2018	Belgium	9:136,339	[13]
2023	Canada (Alberta)	5:47,005	[55]
2020	Canada (Ontario)	5:139,810	[15]
2018	China	3:29,364	[56]
2018	Germany	43:297,163	[16]
2019	Italy	15:90,885	[17]
2020	Japan	0:22,209	[12]
2021	Japan (Osaka)	0:10,000	[57]
2021	Latvia	2:10,411	[18]
2023	Portugal	2:25,000	[58]
2019	Russia	3:23,405	[19]
2014	Taiwan	7:120,267	[59]
2016	USA	180:2,395,718	[14]

Pilot studies conducted between 2014 and December 2023 in 14 countries with established 5q-SMA local incidence.

#### 5q-SMA NBS and Its Context within the Public Health System in Brazil

In Brazil, the Ministry of Health, supported by the National Commission for the Incorporation of Technologies (Conitec) in the Unified Health System (SUS), is tasked with the “inclusion, exclusion, or alteration of new medicines, products, and procedures, as well as the establishment or modification of clinical protocols or therapeutic guidelines”. Remarkable aspects such as scientific evidence (effectiveness, accuracy, efficiency, and safety of the technology), economic evaluation, and budgetary impact assessment are the core structure of Conitec reports that follow an open public consultation. With a favorable recommendation from Conitec and acceptance by the Ministry of Health, a new treatment, product, or procedure is made publicly available in the SUS [60].

**Figure 3:**
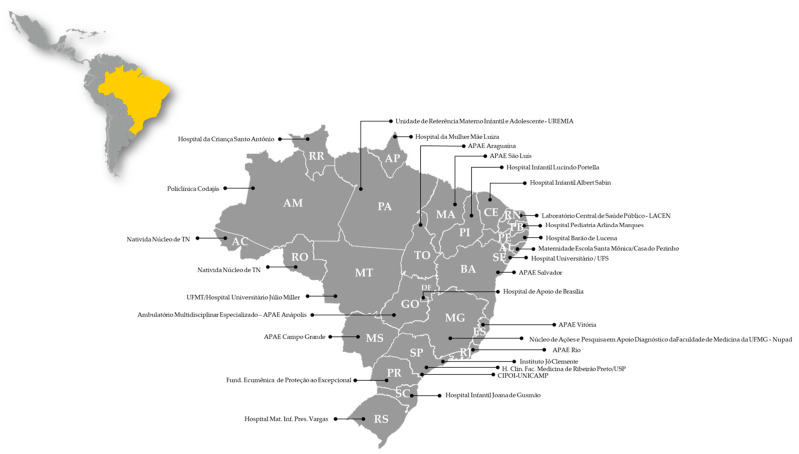
Neonatal Screening Services in Brazil. Brazil is depicted in yellow in the upper left figure and is maximized for detailed visualization. Each state hosts one or more institutions accredited by the Ministry of Health to conduct NBS. AC—Acre; AL—Alagoas; AM—Amazonas; AP—Amapá; BA—Bahia; CE—Ceará; DF—Distrito Federal; ES—Espírito Santo; GO—Goiás; MA—Maranhão; MG—Minas Gerais; MS—Mato Grosso do Sul; MT—Mato Grosso; PA—Pará; PB—Paraíba; PE—Pernambuco; PI—Piauí; PR—Paraná; RJ—Rio de Janeiro; RN—Rio Grande do Norte; RO—Rondônia; RR—Roraima; RS—Rio Grande do Sul; SC—Santa Catarina; SE—Sergipe; SP—São Paulo; TO—Tocantins.

Additionally, in Brazil, the Ministry of Health has established reference services in newborn screening across the country. All states have one reference service in NBS, but São Paulo is the only one to have three: Instituto Jô Clemente (IJC), Hospital das Clínicas da Faculdade de Medicina de Ribeirão Preto (HCFMRP-USP), and Centro Integrado de Pesquisas Oncohematológicas na Infância (CIPOI)-UNICAMP (Figure 3). The IJC is the largest neonatal screening laboratory in Latin America, conducting over 2.7 million tests in 2022 and covering 64% of the state’s SUS NBS tests [22,61]. The coverage in São Paulo state is followed by CIPOI-UNICAMP and HCFMRP-USP with 21% and 15%, respectively. Together, the reference services in NBS play a crucial role in establishing the workflow for patients with altered results, ensuring prompt diagnostic confirmation, and facilitating early contact between patients and physicians [61,62].

So far, only one pilot study has been published for 5q-SMA NBS in the Brazilian population with the aim to validate a screening method in a real sample population of *Rio Grande do Sul* and *São Paulo* states. The study reports four 5q-SMA-confirmed patients among 40,000 screened newborns. An incidence is estimated in 1:10,000 live births [63]. Although the melt curve protocol has shown good accuracy in the study, some challenges might be faced in an automated setting and large-scale analysis, depending on the number of samples to be screened at each center [22].

To gather more comprehensive indicators crucial for governmental policies, IJC is conducting a 5q-SMA NBS Pilot Study in the São Paulo state, aiming to screen 192,000 individuals by May 2025 [64]. The ongoing study at IJC is being developed through a robust partnership involving the State and City Health Departments of São Paulo, in collaboration with CIPOI-UNICAMP, HC-FMRP/USP, and six specialized medical centers focusing on 5q-SMA and/or neuromuscular diseases to the referral of positive patients (personal communication, Romanelli Tavares, 2023). Collectively, this collaboration in São Paulo state underscores the importance of a structured government approach and essential stakeholders to initiate a pilot program that aims to transition into a national program and to serve as a model for other states.

### 3.3. Brazilian Algorithms for 5q-SMA in a Newborn Screening Context

Given the overall context of 5q-SMA NBS, reference services for NBS must align their workflow to address new requirements. This involves significant changes, such as reaching out to support laboratories or making investments in infrastructure and equipment to conduct molecular tests, such as real-time qPCR for 5q-SMA screening, along with simultaneous screening for Severe Combined Immunodeficiency (SCID) and Agammaglobulinemia (also included in the approved federal law) [22,46]. While exploring the needs, existing solutions, and the current Clinical Protocols and Therapeutic Guidelines applicable within the Brazilian UHS, the IJC, and collaborators of the ongoing São Paulo state 5q-SMA NBS Pilot Study have developed a local white paper regarding the local context of 5q-SMA NBS. The document was titled “*Guidance for the diagnosis, follow-up, and treatment of patients with pre-symptomatic 5q spinal muscular atrophy (5q SMA) identified through newborn screening*” (English translation; original text only available in Portuguese) [64]).

#### 3.3.1. Molecular Tests

In Brazil, blood on filter paper collection occurs, preferably, immediately after 48 h and 1 min for all NBS tests currently performed. On average, the first sample collection occurs between the third and fifth days of a child’s life [65]. The screening results are expected to be released as soon as possible, with some states performing it within 7 business days after the sample arrives. In the case of an altered result, the parents should be promptly informed by the active search service of the reference service to collect a new sample for the confirmatory test.

In the case of 5q-SMA NBS, the São Paulo state pilot study has been conducting a qualitative real-time PCR test (first tier) and employing the MLPA for *SMN1*/*SMN2* as a second-tier test (data to be published-personal information Romanelli Tavares 2014). Concerned with early cases of 5q SMA, alongside the confirmatory test sample collection, the newborn found altered at the first-tier test is promptly referred to a specialized center for a comprehensive clinical evaluation to identify and address any potential symptoms requiring specific interventions, such as respiratory care. It is noteworthy that a patient with an abnormal screening test (lack of *SMN1* exon 7) and classic symptoms of early 5q-SMA may require immediate interventional therapy upon clinical decision. Both symptomatic and asymptomatic patients undergo a second clinical evaluation after the release of MLPA results, which complements treatment decisions based on the evaluation of *SMN2* copy number. It is also important to highlight that some services may be able to perform the confirmatory test using the sample already collected on the paper filter. This is possible with a standardized protocol for DNA extraction followed by MLPA for *SMN1* and *SMN2* [65].

When considering confirmatory testing, while MLPA has traditionally been deemed the gold standard for diagnosing 5q-SMA and is recommended as the confirmatory test for positive cases detected through NBS, the use of RFLP presents itself as a potential alternative to expedite results, as outlined in Mikhalchuk et al. [19]. However, RFLP cannot assess the copy number of *SMN2*, so the completion of MLPA is needed for accurate evaluation and, in some instances, as a treatment guide. On the other hand, MLPA technology encounters limitations when quantifying more than 3 *SMN2* copies. Some studies have turned to digital PCR [66,67], which has shown superior precision in copy number determination compared to MLPA [68]. Two important studies have evaluated the accuracy of MLPA by comparing results across different laboratories or by performing digital PCR [69,70]. Assessing MLPA results at different times and between two different laboratories revealed a 45% discrepancy when compared to the initial *SMN2* copy number result [70]. In a comparison between MLPA and digital PCR (n = 733 DNA samples), Jiang and collaborators [69] identified 41 (95.6%) results with discrepancies in *SMN1* and/or *SMN2* copy numbers; 37 of these 41 discrepancies were attributed to MLPA inaccuracy. It is important to highlight that the determination of *SMN2* copy number is crucial for treatment recommendations [71]. Germany itself reports that the *SMN2* copy number is crucial for therapeutic selection, as gene replacement therapy is only available for patients with 2 or 3 *SMN2* there [50]. It is crucial to note that regardless of the confirmatory test chosen for the 5q-SMA NBS program in Brazil, the limited demand for confirmatory tests might be suitable for performance in supportive laboratories, providing a more cost-effective solution.

**Figure 4:**
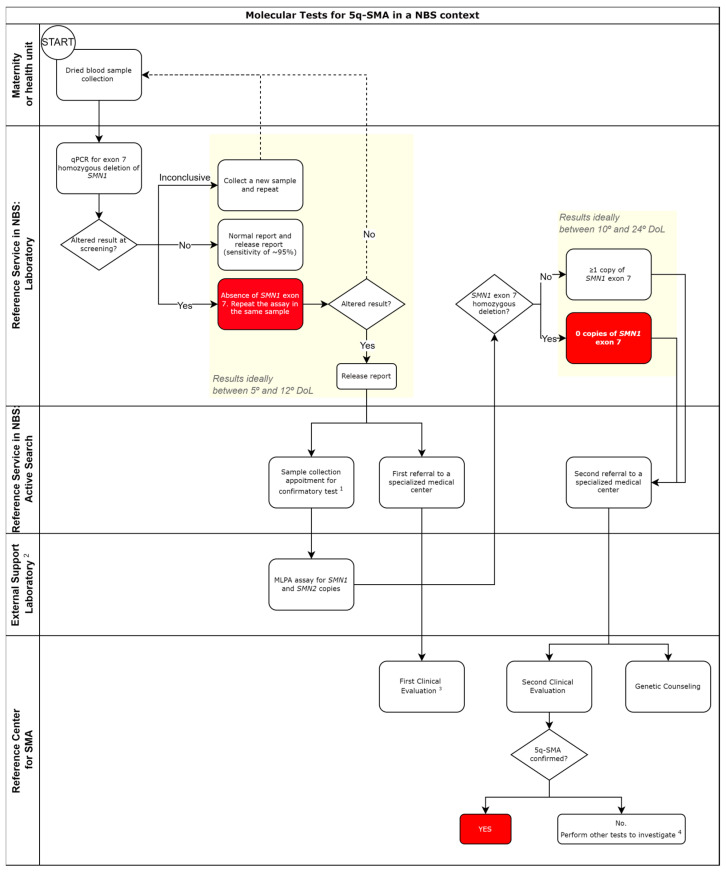
Scheme of genetic tests for newborn screening of 5q-SMA using qualitative testing, followed by confirmatory quantitative testing. Legend: *SMN1*—survival motor neuron 1 gene; qPCR—Real-Time Polymerase Chain Reaction; MLPA—Multiplex Ligation-dependent Probe Amplification. 1—The confirmatory test (MLPA) can be performed using the paper filter sample [67]. 2—For laboratories not able to implement confirmatory tests (usually due to low demand), tests can be performed by an external service. 3—The first clinical evaluation is highly recommended because patients showing early symptoms of the disease can receive adequate treatment. 4—Some cases of 5q-SMA may present with one or two alleles with subtle variants (small deletions/insertions or single nucleotide variants); Sanger or Next-Generation Sequencing (NGS) can identify such variants but not always distinguish the *SMN1*/*SMN2* paralogous genes. Adapted from [64].

Furthermore, genetic counseling is strongly recommended and, in the São Paulo Pilot Study, being provided at the NBS reference center. It aims to assist with family planning and offer emotional support to parents, facilitated by a social worker if necessary. The recommended workflow for molecular assays in an NBS context is presented in Figure 4.

#### 3.3.2. Genetic Counseling

In general, genetic counseling as a program has been implemented only in a few countries worldwide. For this reason (and ethics concerns), the quantitative screening tests for *SMN1* copy numbers have been limited to some countries, such as New York [66,72], as they also identify carrier patients. Although the first two Brazilian Pilots used qualitative or semi-quantitative 5q-SMA NBS tests [22,73], the importance of genetic counseling remains indispensable after the confirmatory test has been completed for the identified newborn and recommended to his/her parents.

Information acquired through NBS plays a critical role in assessing the recurrence risk within the child’s family, prompting recommendations for carrier testing and counseling among parents, siblings, and close relatives. The diagnostic approach for relatives is contingent on the variant identified in the initial patient, with methodologies such as NGS potentially complementing MLPA in about 1.7% of parent’s cases [74,75]. Specifically for parents, it is also important to consider silent carriers, depending on the result of the investigation. A silent carrier is characterized by an *SMN1* deletion on one chromosome and two *SMN1* copies on the other, which is a result that seems like parents presenting with 2 *SMN1* copies. However, traditional quantitative methods like MLPA do not detect them, requiring alternate approaches like target PCR for specific variants followed by capillary electrophoresis fragment analysis [76]. Moreover, de novo deletions (2%) and germline mosaicism might also explain a child affected by the 5q-SMA with one of the parents presenting a normal genotype [77,78].

**Figure 5:**
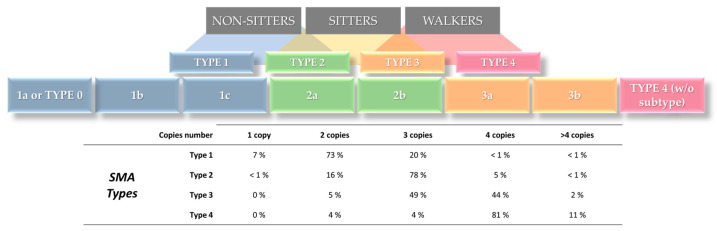
5q-SMA Classification and correlation with *SMN2* copy numbers. The figure above illustrates the new classification of patients with 5q-SMA into three main groups: NON-SITTERS, SITTERS, and WALKERS. This classification was proposed with the advent of new therapeutic options to ensure consistency in clinical follow-up. The original classification of 5q-SMA types is also presented in the figure, as types 1, 2, 3, and 4 and their subtypes ranging from the most clinically severe, type 1a or type 0, to the mildest in symptoms and severity, type 4. It is important to note that there is overlap among the 5q-SMA types encompassed by the new and original classification. Below the disease classification, the distribution, in percentage, of the number of *SMN2* copies affecting each type of 5q-SMA has been described (adapted from and reviewed in [74]).

In pregnancies with both parents as carriers, there is a 25% risk of 5q-SMA recurrence. However, predicting the severity of the condition and its variation between siblings remains a challenging task [79]. Prognostic information should be disclosed cautiously by qualified professionals due to the lack of absolute correlation between *SMN2* gene copies and disease severity (Figure 5). This careful approach ensures that families and caregivers comprehend and manage the situation accurately [80].

#### 3.3.3. Clinical Evaluation and Follow-Up

Clinical assessment is of fundamental importance in identifying patients who are truly asymptomatic or have minimal initial manifestations. This analysis becomes especially crucial in extremely severe cases, where newborns may present respiratory compromise in the first days of life. If possible, specialists in neuromuscular/neuropediatric should carry out the initial clinical assessment, even in neonatal intensive care units. In regions with limited neuromuscular specialists, pediatricians/neonatologists can guide the diagnosis, referring patients to more specialized centers.

In 2019, a resolution (SS 89) was published in the Official Gazette of the state of São Paulo, establishing a working group to address matters concerning 5q SMA. This resolution was accessed to invite and include specialized professionals in the pilot study for 5q SMA NBS. The collaboration between the IJC and the NBS program coordinator of São Paulo state allowed this inclusion. The group, informally known as “Specialized Reference Centers for Spinal Muscular Atrophy”, or “CERAMEs” (in Portuguese—Centro Especializado de Referência para a Atrofia Muscular Espinhal), is composed of eight specialized physicians of seven renowned hospitals in the São Paulo state. Using this model, our expectation is that other states in Brazil can establish and work with similar groups, ensuring the appropriate referral of identified 5q-SMA patients in an NBS program [81].

As described, identified newborns went to at least two clinical evaluations after a first-tier altered result. A clinical evaluation must include assessing neuromuscular manifestations such as hypotonia, reduced or absent antigravity movements, tongue fasciculations, diminished/absent deep tendon reflexes, and respiratory symptoms like bell-shaped thorax and paradoxical breathing [9]. Clinical signs that may indicate 5q-SMA type 0 must also be of note, such as the presence/absence of intrauterine movements, deformative sequence due to fetal akinesia, cardiac anomalies, and quadriparesis [82].

Furthermore, motor scale assessments, through CHOP-INTEND, of 5q-SMA patients identified by NBS are highly valued. It is important to note that Brazil does have a Brazilian Portuguese version of CHOP-INTEND, which has been translated and validated, ensuring adherence to quality criteria in evaluations and enabling its utilization in a more accessible manner for the local context [83]. Monitoring at specialized centers is recommended with an interdisciplinary team (neurologists, pediatricians, geneticists, physiotherapists, speech therapists, and nutritionists), especially with the inclusion of disease-modifying therapies.

**Figure 6:**
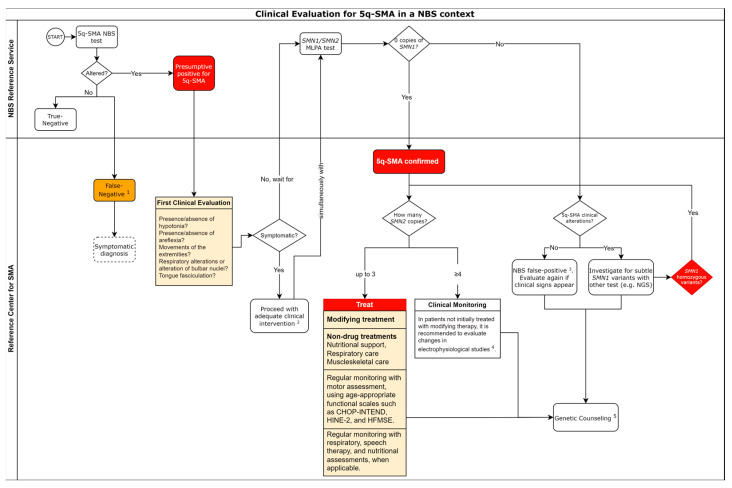
Clinical Approach in the Case of Suspected 5q-SMA Due to Abnormal Newborn Screening Test. Currently, pre-symptomatic patients are included in the Clinical Protocols and Therapeutic Guidelines (Portuguese: Protocolos Clínicos e Diretrizes Terapêuticas; PCDT) with limited criteria; these should be reviewed as new studies are concluded, and ANVISA approvals are obtained. Electrophysiological assessment is recommended for detecting denervation of muscle fibers, and its application should be considered based on medical expertise and patient comfort regarding an assessment for early disease detection. NBS—newborn screening. SMA—Spinal muscular atrophy. *SMN1*—survival motor neuron 1 gene; *SMN2*—survival motor neuron 2 gene; MLPA—Multiplex Ligation-dependent Probe Amplification. NGS—next-generation sequencing. CHOP-INTEND (Children’s Hospital of Philadelphia Infant Test of Neuromuscular Disorders); HINE-2—Hammersmith Infant Neurological Exam-Part 2; HFMSE—Hammersmith Functional Motor Scale Expanded. Adapted from [64]. 1—False-negative results are expected to occur in about 5% of screened newborns. 2—Adequate clinical intervention can include non-invasive ventilation, nutritional support, or disease-modifying therapy. 3—False-positive results can occur due to polymorphisms in the primer or probe region. 4—Electrophysiological evaluation is recommended for detecting muscle fiber denervation, and its application should be considered based on medical expertise and patient comfort in relation to an assessment for detecting the onset of the disease. 5—Genetic counseling can be offered either in the reference center for SMA or the NBS Reference Service when possible.

Alternatively, if the patient is not eligible for immediate treatment (as will be further discussed), regular neuromuscular assessments concentrating on strength, reflexes, and fasciculation should be conducted. The initiation of treatment with 5q-SMA modifying drugs is recommended at the earliest indication of symptoms. Monitoring in this scenario involves examining the compound muscle action potential (CMAP) of the ulnar and common fibular nerve, which values below 80% or any signs of denervation indicate early disease onset. In the absence of such indicators, children undergo evaluation every 3 to 12 months initially, transitioning to assessments every 6 to 12 months starting at age 2. Additionally, the motor unit number index (MUNIX) has been highlighted as a valuable biomarker for measuring motor unit loss in 5q-SMA patients with later onset [84]. Surveillance is conducted by pediatric neurologists, while physical therapists are encouraged to employ CHOP-INTEND for evaluations up to 2 years of age or >2 years old for patients without the ability to sit. Subsequently, regular HFMSE assessments are advised for children older than 2 years with the ability to sit, with 6MWT and RULM examinations commencing at age 3 and 2, respectively [85]. An objective representation of the recommendations described can be seen in Figure 6.

Although the NBS expects to mostly identify pre-symptomatic newborns, some symptomatic patients might be found, as demonstrated by Boemer et al. and Hale et al. [13,86]. In such cases, it is essential to adhere to well-established recommendations for clinical care, which involve (1) managing pulmonary complications, (2) providing nutritional and gastrointestinal support, (3) offering orthopedic care, and (4) implementing rehabilitation interventions as needed [85,87]. Specialized attention to the respiratory system is crucial, especially due to the range of respiratory severity phenotypes, requiring daily care to improve airway clearance and evaluate and treat bulbar dysfunction, pulmonary aspiration, and lower respiratory tract infections [86,88]. It is of utmost importance to bear in mind that even in the context of modifying therapy administration, interdisciplinary attention should continue to focus on stimulating patient functionality and improving quality of life. In this way, it is expected that the patient’s phenotype may stabilize or even improve [85].

#### 3.3.4. Disease-Modifying Therapies

In Brazil, ANVISA-approved drugs for 5q-SMA treatment include Nusinersen, Risdiplam, and Onasemnogene abeparvovec (Table 2). Currently, the simultaneous use of these medications is not recommended in Brazilian clinical protocols due to the lack of published scientific evidence demonstrating safety and clinical benefits [85,89]. One publication studying the administration of both therapies in seven patients suggests that early treatment might be more beneficial than combined therapy [90,91,92]. Additional studies need to be conducted to further clarify this.

One important thing to be carefully evaluated by the specialist, regardless of the chosen therapeutic approach, is the number of *SMN2* copies and clinical characteristics to guide treatment decisions. The international medical and scientific community, along with the implementation of the 5q-SMA NBS in several countries, emphasizes the urgency of providing immediate access to disease-modifying therapy for infants with up to four copies of *SMN2* (per international consensus) [72]. However, the Brazilian indication is yet limited to three *SMN2* copies [89]. Prompt determination of *SMN2* copy number enhances the decision-making process for the treatment and management of SMA detected in a newborn screening program. If possible, *SMN2* quantification should be performed concurrently with or in a timely manner following *SMN1* screening [66,67,71]. Timely and effective therapeutic interventions are vital to prevent the irreversible loss of motor neurons, even before the onset of any clinical symptoms [9].

Considering the presence of four *SMN2* copies in a child diagnosed with 5q-SMA through NBS, the patient must be monitored for symptoms onset, as aforementioned described. While most children (81%) with four *SMN2* copies tend to develop 5q-SMA type 4, in about 5% of the cases, the patients develop as type 2. Hence, any indication of symptoms onset during neuromuscular examinations determines the immediate initiation of disease-modifying therapy as internationally recommended [71,94].

#### 3.3.5. Clinical Protocols and Therapeutic Guidelines (Portuguese: *Protocolos Clínicos e Diretrizes Terapêuticas, PCDT*)

PCDTs are documents that establish criteria for the diagnosis, treatment, monitoring, and clinical control of diseases within the Unified Health System, SUS, in Brazil. It is important to highlight that the SUS aims to offer full, universal, and free access to healthcare services, from simple to complex ones, including therapies, to the entire country. Based on scientific evidence, these protocols guide healthcare managers on procedures and therapies, considering the effectiveness, safety, and cost-effectiveness of the recommended approaches. As aforementioned, once Conitec and the Ministry of Health approve the inclusion of a new therapy in the SUS, establishing the respective PCDT, it is made available in the public health system. In the case of 5q SMA, the PCDT describes modifying therapies and interdisciplinary treatment such as nutritional, respiratory, and orthopedic care, addressing the multifaceted challenges associated with the disease.

The 5q SMA modifying treatments included in the SUS are Risdiplam and Nusinersen. Costs regarding these treatments have been covered by SUS. Despite the fact that Onasemnogen abeparvovec has been recommended to be included in the SUS in December 2022, it is not yet available to patients (see Table 2). The proposal for incorporating it centers around a risk-sharing agreement between the Brazilian federal government and the pharmaceutical company. In summary, under this type of agreement, which is already practiced in other countries, payment for the medication would be made in installments based on the achievement of clinical parameters established by the government, such as overall survival, ventilator-free survival, and attainment of motor milestones. These milestones would be evaluated over a period of five years and were made due to the long-term efficacy uncertainties highlighted by Conitec [93].

Moreover, in Brazil, the current PCDT for 5q-SMA remains focused on patients classified as type 1 and type 2, a classification delineated by clinical onset and motor achievements according to the literature [89]. This version of the PCDT mentions the inclusion of pre-symptomatic patients but confines eligibility to those with a familial history of 5q-SMA, genetic confirmation, and up to 3 copies of *SMN2* within type 1 and type 2. However, the PCDT lacks clarity on how disease classification is determined in pre-symptomatic cases, given their absence of disease signs; presumably, classification relies on *SMN2* copy number predictions.

A preliminary update to the 5q-SMA PCDT, aiming to incorporate Onasemnogene abeparvoveque into the document while revising the indications for Risdiplam and Nusinersen (previously included), underwent public consultation in September 2023 [95]. An advancement noted in this preliminary update concerns the inclusion of pre-symptomatic patients without having a familial history of 5q-SMA (despite being described as type 1 and 2) for potential access to modifying drugs through SUS. Although not aiming at the NBS, this modification signifies a crucial step toward the anticipated inclusion of 5q-SMA in an NBS program. It is important to highlight that being a preliminary version means that it may be subject to change after public consultation.

## 4. Discussion

It has been over 60 years since the inception of newborn screening, and it has had great success worldwide. NBS programs constitute a holistic framework that includes education, screening, diagnosis, referral, short-term follow-up, treatment, care management, long-term follow-up, and ongoing assessment of the effectiveness of all components.

Ideally, education about newborn screening should commence during the prenatal period, with obstetricians providing information to expectant parents and, later, the pediatricians. It is crucial for both the healthcare system and parents to actively engage and be dedicated to initiating the screening process for infants, whether at the hospital, birthing facility, or healthcare units, and to pursue referrals for late collection or repetition at a reference service, if necessary [96,97].

Even though rapid advances in neonatal health have been developed, they cannot be said to be equitable across the world, and the uneven global implementation of newborn molecular screening transcends local challenges. Historically, the LAC region has had issues concerning complex infrastructure requirements and insufficient data for informed implementation of NBS, especially genetic NBS. Predominantly, pilot studies have been conducted in high-incoming countries, which brings a bias and poor representativeness when talking about low- and middle-income countries [98].

For instance, considering the 5q-SMA NBS, a lack of studies conducted in the LAC is evident in the scientific literature. A recent systematic review regarding the treatment of patients identified by newborn screening reports data from the United States, Asia, Europe, Australia, Canada, and the LAC. Despite the first 5q-SMA modifying drug, Nusinersen, being approved in Brazil in 2017 and first included in the SUS in 2019, the 5q-SMA NBS has just started in some states, such as Minas Gerais and Distrito Federal (Brasília) via government implementation, with no data published. On the other hand, research pilot studies have been conducted in the state of Rio Grande do Sul [63] and another one in São Paulo [personal communication Romanelli Tavares, data to be published]. Taken together, 4 out of 26 states have been conducting the 5q-SMA NBS somehow.

Most of the worldwide routine practice in 5q-SMA NBS reports the use of qualitative real-time PCR, followed by MLPA to confirm and assess the copy number of *SMN1* and *SMN2* [74]. The 5q-SMA screening via qPCR typically covers approximately 95% of patients, but the remaining 5% may yield false negatives due to compound heterozygotes (with an *SMN1* exon 7 deletion in one allele and a subtle variant in the other allele) or homozygotes carrying alterations beyond the exon 7 deletion in *SMN1*. Notably, in a Brazilian study examining 402 symptomatic patients, 48 individuals (10.7%) exhibited compound heterozygous variants in *SMN1* [99]. Although based on very specific sampling, one might care for the potential higher prevalence of such genotypes in the Brazilian population, raising the number of false-negative results at screening.

Compared to the pilot study performed in Massachusetts [100], the current São Paulo state pilot also includes an initial clinical evaluation of the newborn immediately after the screening test, allowing the identification of newborns with early symptoms of the disease and, if necessary, the initiation of appropriate therapy. This could represent an important step, especially considering that confirmatory testing may take from 8 to 20 days, depending on the Brazilian region. The Brazilian pilot is going to assess the urgency of treatment in some patients identified with NBS further. Identifying early symptoms of the disease is crucial to establish better clinical conduction and may define a better prognosis, as some newborns might present with respiratory impairment in the first days of life [101]. Aragon-Gawinska et al. [8] revisited 13 follow-up studies of 5q-SMA patients identified in the NBS. Several issues were identified as the cause of delay in diagnosis, delay or no treatment, and even death of untreated patients: reimbursement and logistic problems, false-negative results, human errors, incorrect quantification of *SMN2*, and parental refusal to treatment. Among treated patients, the mean age at treatment was 23 days, 52, and 219 days for 5q-SMA patients with 2, 3, or 4 *SMN2* copies. Patients with 2 copies of the *SMN2* gene exhibited symptoms upon treatment initiation in 51% of cases, whereas only 2% and 25% of those with 3 and 4 copies of *SMN2*, respectively, did so.

It is important to highlight that not all concerns regarding the treatment of 5q-SMA patients identified in the NBS are considered in the current clinical guidelines (PCDT) in Brazil. The document focuses on patients classified as type 1 and type 2 even in the pre-symptomatic context, a classification delineated by clinical onset and motor achievements [4]. Therefore, the PCDT remains to clarify how the disease is classified in non-symptomatic patients; presumably, classification relies on *SMN2* copy number predictions. Moreover, discussions regarding whether to wait or treat patients (in the SUS) with 4 *SMN2* copies identified in the NBS should take place. These discussions should consider themes such as the high cost of therapy, societal burden, safety, and the need for real-world evidence, which is still pending further research [16]. The consensus from the Brazilian Academy of Neurology sheds light on some of these topics [85].

In summary, to the best of our knowledge, this work provides a pioneering reference for the LAC region on how to conduct the end-to-end flow for identifying 5q-SMA patients in an NBS context. Local adaptations of this model should be made to best suit the specific needs of each region. Additionally, it is crucial to account for pre-symptomatic patients and individuals with more than 3 copies of *SMN2* in an NBS context.

## Figures and Tables

**Table 2:** Modifying therapies for 5q-Spinal Muscular Atrophy (SMA) and their respective approvals and indications. Non-drug treatments are not considered here.

Drug Name	Mechanism of Action	Administration	Label Information: Doses	Brazilian Regulation (ANVISA)	Label Information: Indication (ANVISA)	Brazilian Academy of Neurology Consensus: Disease-Modifying Therapies in 5q-SMA ^1^	Brazilian Clinical Protocol (PCTD) in the Public Health System (SUS) Inclusion Criteria of Preliminary Version ^2^
Nusinersen (SPINRAZA^TM^)	Anti-sense oligonucleotide that enables the inclusion of exon 7 during the processing of SMN2 mRNA, transcribed from DNA (*SMN2* gene). Retention of exon 7 in SMN2 mRNA allows for the correct reading and translation of this molecule, leading to the production of functional SMN protein, which is related to motor neuron survival.	Intrathecal administration	12 mg (5 mL) per administration. Loading dose phase: First three doses every 14 days (on days 0, 14, and 28). The fourth dose should be administered 30 days after the third. Maintenance phase: Every four months.	Yes	To treat patients with 5q-SMA (pediatric and adult).	Effectiveness and safety demonstrated for the treatment of pre-symptomatic patients and those with 5q-SMA types 1, 2, and 3.	5q-SMA type 1: Pre-symptomatic patients with genetic diagnosis and up to 3 copies of *SMN2*; Symptomatic patients with genetic diagnosis, up to 3 copies of *SMN2*, and symptom onset up to the sixth month of life. 5q-SMA type 2: Pre-symptomatic patients with genetic diagnosis and up to 3 copies of *SMN2*; Symptomatic patients with genetic diagnosis, up to 3 copies of *SMN2*, symptom onset between 6 and 18 months of age, and: be up to 12 years old at the start of treatment OR over 12 years of age at the beginning of treatment and preserved ability to sit without support and upper limb function.
Risdiplam (EVRYSDI^®^)	Splicing modifier of pre-mRNA from the survival motor neuron 2 (*SMN2*) gene, to shift the balance from exon 7 exclusion to its inclusion in the transcribed mRNA, promoting an increase in the production of stable and functional SMN protein.	Oral administration	Daily dose, adjusted based on age and body weight.	Yes	To treat patients with 5q-SMA (pediatric and adult).	Effectiveness and safety demonstrated for the treatment of pre-symptomatic patients and those with 5q-SMA types 1, 2, and 3.	5q-SMA type 1: Pre-symptomatic patients with genetic diagnosis and up to 3 copies of *SMN2*; Symptomatic patients with genetic diagnosis, up to 3 copies of *SMN2*, and symptom onset up to the sixth month of life. 5q-SMA type 2: Pre-symptomatic patients with genetic diagnosis and up to 3 copies of *SMN2*; Symptomatic patients with genetic diagnosis, up to 3 copies of *SMN2*, symptom onset between 6 and 18 months of age, and: be up to 12 years old at the start of treatment OR over 12 years of age at the beginning of treatment and preserved ability to sit without support and upper limb function. NOTE that an important limitation for Risdiplam is exclusion of patients under 16 days of life, due to loss of safety and eficacy evidences.
Onasemnogen abeparvovec (ZOLGENSMA^®^)	Recombinant gene therapy based on AAV9, developed to deliver a copy of the gene encoding human SMN protein.	Intravenous infusion administration	1.1 × 10^14^ vector genomes per kilogram (vg/kg) of body weight. Single dose.	Yes	Pediatric 5q-SMA patients, under 2 years of age, with: bi-allelic mutations in *SMN1* and 5q-SMA type 1; OR bi-allelic mutations in *SMN1* and up to 3 copies of *SMN2* ^3^.	Effectiveness demonstrated for the treatment of pre-symptomatic patients and those with 5q-SMA type 1 up to 6 months of age. Also for patients with types 1 and 2, aged up to 24 months and a body weight of up to 13.5 kg. Safety has not been demonstrated for patients older than 2 years or more than 13.5 kg.	Pediatric patients up to 6 months of age with 5q-SMA type 1 who are not on invasive ventilation for more than 16 hours per day and with up to 3 *SMN2* copies.

mRNA—Messenger RNA (ribonucleic acid); AAV9—adeno-associated viral vector serotype 9; *SMN2*—survival motor neuron 2 gene; SMN—survival motor neuron protein; PCDT—Clinical Protocols and Therapeutic Guidelines; SUS—Unified Health System; CONITEC—National Committee for the Incorporation of Technologies in the Unified Health System. ANVISA—National Health Surveillance Agency; FDA—Food and Drug Administration. ^1^ Zanoteli, et al. [85]; ^2^ Preliminary Recommendation Report, based on the 122nd Ordinary Meeting of CONITEC, held on 14 September 2023 [93]; the document may suffer changes. ^3^ Different from ANVISA, in Brazil, the FDA label indication does not restrict the the use of Zolgensma^®^ to SMA type neither to *SMN2* copy number. The limitation is just by age under 2 years.

## Data Availability

The original contributions presented in the study are included in the article/Supplementary Material, further inquiries can be directed to the corresponding authors.

## Supplementary Materials

The following supporting information can be downloaded at: https://www.mdpi.com/article/10.3390/genes15070858/s1, Table S1: Evidence search strategies in databases.
