# Supplementary material for: Integrated Approaches and Practical Recommendations in Patient Care Identified with 5q Spinal Muscular Atrophy through Newborn Screening
Source: Genes (Basel). 2024 Jun 29;15(7):858. doi: 10.3390/genes15070858 (PMC11276409; doi:10.3390/genes15070858)
Supplement: Supplementary file 1 [file genes-15-00858-s001.zip › genes-3054385-supplementary.pdf]

Supplemental Material S1. Evidence search strategies in databases.

Database	Search strategies	Number of articles (on sep14 2022)
Embase	#1<br>'spinal muscular atrophy'/exp OR 'adult onset spinal muscular atrophy':ti,ab,kw OR 'adult spinal muscular atrophy':ti,ab,kw OR 'adult-onset spinal muscular atrophy':ti,ab,kw OR 'amyotrophies, spinal':ti,ab,kw OR 'amyotrophy, neurogenic scapuloperoneal, new england type':ti,ab,kw OR 'amyotrophy, spinal':ti,ab,kw OR 'atrophies, progressive muscular':ti,ab,kw OR 'atrophy, myelopathic muscular':ti,ab,kw OR 'atrophy, progressive muscular':ti,ab,kw OR 'atrophy, spinal muscular':ti,ab,kw OR 'bulbospinal neuronopathies':ti,ab,kw OR 'bulbospinal neuronopathy':ti,ab,kw OR 'distal spinal muscular atrophy':ti,ab,kw OR 'hereditary motor neuronopathies':ti,ab,kw OR 'hereditary motor neuronopathy':ti,ab,kw OR 'motor neuronopathies, hereditary':ti,ab,kw OR 'motor neuronopathy, hereditary':ti,ab,kw OR 'muscular atrophies, progressive':ti,ab,kw OR 'muscular atrophy, adult spinal':ti,ab,kw OR 'muscular atrophy, myelopathic':ti,ab,kw OR 'muscular atrophy, progressive':ti,ab,kw OR 'myelopathic muscular atrophy':ti,ab,kw OR 'myelopathic muscular atrophy, progressive':ti,ab,kw OR 'neuronopathies, bulbospinal':ti,ab,kw OR 'neuronopathies, hereditary motor':ti,ab,kw OR 'neuronopathy, bulbospinal':ti,ab,kw OR 'neuronopathy, hereditary motor':ti,ab,kw OR 'oculopharyngeal spinal muscular atrophy':ti,ab,kw OR 'progressive muscular atrophies':ti,ab,kw OR 'progressive muscular atrophy':ti,ab,kw OR 'progressive myelopathic muscular atrophy':ti,ab,kw OR 'progressive proximal myelopathic muscular atrophy':ti,ab,kw OR 'proximal myelopathic muscular atrophy, progressive':ti,ab,kw OR 'scapuloperoneal form of spinal muscular atrophy':ti,ab,kw OR 'scapuloperoneal spinal muscular atrophy':ti,ab,kw OR 'spinal amyotrophies':ti,ab,kw OR 'spinal amyotrophy':ti,ab,kw OR 'spinal muscular atrophy':ti,ab,kw OR 'spinal muscular atrophy, distal':ti,ab,kw OR 'spinal muscular atrophy, oculopharyngeal':ti,ab,kw OR 'spinal muscular atrophy, scapuloperoneal':ti,ab,kw OR 'spinal muscular atrophy, scapuloperoneal form':ti,ab,kw OR 'hereditary spinal muscular atrophy'/exp OR 'spinal muscular atrophies of childhood':ti,ab,kw OR 'infantile muscular atrophy':ti,ab,kw OR 'infantile spinal muscular atrophy':ti,ab,kw OR 'juvenile muscular atrophy':ti,ab,kw OR 'juvenile spinal muscular atrophy':ti,ab,kw OR 'kugelberg welander disease':ti,ab,kw OR 'kugelberg welander syndrome':ti,ab,kw OR 'kugelberg-welander disease':ti,ab,kw OR 'kugelberg-welander syndrome':ti,ab,kw OR 'muscular atrophy, infantile':ti,ab,kw OR 'muscular atrophy, juvenile':ti,ab,kw OR 'muscular atrophy, spinal, infantile':ti,ab,kw OR 'muscular atrophy, spinal, infantile chronic form':ti,ab,kw OR 'muscular atrophy, spinal, intermediate type':ti,ab,kw OR 'muscular atrophy, spinal, type iii':ti,ab,kw OR 'sma, infantile acute form':ti,ab,kw OR 'spinal muscular atrophy type iii':ti,ab,kw OR 'spinal muscular atrophy, infantile':ti,ab,kw OR 'spinal muscular atrophy, juvenile':ti,ab,kw OR ('spinal muscular atrophy, mild childhood':ti,ab,kw AND 'adolescent form':ti,ab,kw) OR 'spinal muscular atrophy, type 3':ti,ab,kw OR 'spinal muscular atrophy, type iii':ti,ab,kw OR 'type iii spinal muscular atrophy':ti,ab,kw OR 'werdnig hoffman disease':ti,ab,kw OR 'werdnig hoffmann disease':ti,ab,kw OR 'werdnig-hoffmann disease':ti,ab,kw<br>#2<br>'diagnostic screening programs'/exp OR 'diagnostic screening program':ti,ab,kw OR 'program, diagnostic screening':ti,ab,kw OR 'programs, diagnostic screening':ti,ab,kw OR 'screening program, diagnostic':ti,ab,kw OR 'screening programs, diagnostic':ti,ab,kw<br><br>#3<br>'survival of motor neuron 1 protein'/exp OR ('smn protein':ti,ab,kw AND 'spinal muscular atrophy':ti,ab,kw) OR 'survival motor neuron protein 1':ti,ab,kw OR 'survival of motor neuron 1, telomeric protein':ti,ab,kw	359

	#1 AND #2 AND #3	
MedLine via PubMed	("muscular atrophy, spinal"[MeSH Terms] OR "muscular atrophy spinal"[Text Word] OR "adult onset spinal muscular atrophy"[Text Word] OR "adult spinal muscular atrophy"[Text Word] OR "adult onset spinal muscular atrophy"[Text Word] OR (("muscular atrophy"[MeSH Terms] OR ("Muscular"[All Fields] AND "Atrophy"[All Fields]) OR "muscular atrophy"[All Fields] OR "Amyotrophies"[All Fields] OR "Amyotrophy"[All Fields]) AND "Spinal"[Text Word]) OR (("muscular atrophy"[MeSH Terms] OR ("Muscular"[All Fields] AND "Atrophy"[All Fields]) OR "muscular atrophy"[All Fields] OR "Amyotrophies"[All Fields] OR "Amyotrophy"[All Fields]) AND "Spinal"[Text Word]) OR (("atrophie"[All Fields] OR "Atrophy"[MeSH Terms] OR "Atrophy"[All Fields] OR "atrophied"[All Fields] OR "Atrophies"[All Fields] OR "atrophying"[All Fields]) AND "progressive muscular"[Text Word]) OR (("atrophie"[All Fields] OR "Atrophy"[MeSH Terms] OR "Atrophy"[All Fields] OR "atrophied"[All Fields] OR "Atrophies"[All Fields] OR "atrophying"[All Fields]) AND "myelopathic muscular"[Text Word]) OR (("atrophie"[All Fields] OR "Atrophy"[MeSH Terms] OR "Atrophy"[All Fields] OR "atrophied"[All Fields] OR "Atrophies"[All Fields] OR "atrophying"[All Fields]) AND "progressive muscular"[Text Word]) OR "atrophy spinal muscular"[Text Word] OR ("Bulbospinal"[All Fields] AND "Neuronopathies"[Text Word]) OR "bulbospinal neuronopathy"[Text Word] OR "distal spinal muscular atrophy"[Text Word] OR "hereditary motor neuronopathies"[Text Word] OR "hereditary motor neuronopathy"[Text Word] OR (("Motor"[All Fields] OR "motor s"[All Fields] OR "motoric"[All Fields] OR "motorically"[All Fields] OR "motorics"[All Fields] OR "motoring"[All Fields] OR "motorisation"[All Fields] OR "motorised"[All Fields] OR "motorization"[All Fields] OR "motorized"[All Fields] OR "motors"[All Fields]) AND ("Neuronopathies"[All Fields] OR "Neuronopathy"[All Fields]) AND "Hereditary"[Text Word]) OR (("Motor"[All Fields] OR "motor s"[All Fields] OR "motoric"[All Fields] OR "motorically"[All Fields] OR "motorics"[All Fields] OR "motoring"[All Fields] OR "motorisation"[All Fields] OR "motorised"[All Fields] OR "motorization"[All Fields] OR "motorized"[All Fields] OR "motors"[All Fields]) AND ("Neuronopathies"[All Fields] OR "Neuronopathy"[All Fields]) AND "Hereditary"[Text Word]) OR (("muscular atrophy"[MeSH Terms] OR ("Muscular"[All Fields] AND "Atrophy"[All Fields]) OR "muscular atrophy"[All Fields] OR ("Muscular"[All Fields] AND "Atrophies"[All Fields]) OR "muscular atrophies"[All Fields]) AND "Progressive"[Text Word]) OR (("muscular atrophy"[MeSH Terms] OR ("Muscular"[All Fields] AND "Atrophy"[All Fields]) OR "muscular atrophy"[All Fields]) AND "adult spinal"[Text Word]) OR (("muscular atrophy"[MeSH Terms] OR ("Muscular"[All Fields] AND "Atrophy"[All Fields]) OR "muscular atrophy"[All Fields]) AND "Myelopathic"[Text Word]) OR "muscular atrophy progressive"[Text Word] OR "myelopathic muscular atrophy"[Text Word] OR (("Neuronopathies"[All Fields] OR "Neuronopathy"[All Fields]) AND "Bulbospinal"[Text Word]) OR (("Neuronopathies"[All Fields] OR "Neuronopathy"[All Fields]) AND "hereditary motor"[Text Word]) OR (("Neuronopathies"[All Fields] OR "Neuronopathy"[All Fields]) AND "Bulbospinal"[Text Word]) OR (("Neuronopathies"[All Fields] OR "Neuronopathy"[All Fields]) AND "hereditary motor"[Text Word]) OR ("Oculopharyngeal"[All Fields] AND "spinal muscular atrophy"[Text Word]) OR "progressive muscular atrophies"[Text Word] OR "progressive muscular atrophy"[Text Word] OR "progressive myelopathic muscular atrophy"[Text Word] OR "progressive proximal myelopathic muscular atrophy"[Text Word] OR ("Scapuloperoneal"[All Fields] AND "Form"[All Fields] AND "spinal muscular atrophy"[Text Word]) OR "scapuloperoneal spinal muscular atrophy"[Text Word] OR "spinal amyotrophies"[Text Word] OR "spinal amyotrophy"[Text Word] OR "spinal muscular atrophy"[Text Word] OR "spinal muscular atrophy distal"[Text Word] OR (("muscular atrophy, spinal"[MeSH Terms] OR ("Muscular"[All Fields] AND "Atrophy"[All Fields] AND "Spinal"[All Fields]) OR "spinal muscular atrophy"[All Fields] OR ("Spinal"[All Fields] AND "Muscular"[All Fields] AND "Atrophy"[All Fields])) AND "Oculopharyngeal"[Text Word]) OR "spinal muscular atrophy scapuloperoneal"[Text Word] OR "spinal muscular atrophies of childhood"[MeSH Terms] OR "spinal muscular atrophies of childhood"[Text	234

	Word] OR "infantile muscular atrophy"[Text Word] OR "infantile spinal muscular atrophy"[Text Word] OR "juvenile muscular atrophy"[Text Word] OR "juvenile spinal muscular atrophy"[Text Word] OR "kugelberg welander disease"[Text Word] OR "kugelberg welander syndrome"[Text Word] OR "kugelberg welander disease"[Text Word] OR "kugelberg welander syndrome"[Text Word] OR "muscular atrophy infantile"[Text Word] OR "muscular atrophy juvenile"[Text Word] OR (("spinal muscular atrophies of childhood"[MeSH Terms] OR ("Spinal"[All Fields] AND "Muscular"[All Fields] AND "Atrophies"[All Fields] AND "Childhood"[All Fields]) OR "spinal muscular atrophies of childhood"[All Fields] OR ("Muscular"[All Fields] AND "Atrophy"[All Fields] AND "Spinal"[All Fields] AND "Infantile"[All Fields])) AND "chronic form"[Text Word]) OR (("muscular atrophy, spinal"[MeSH Terms] OR ("Muscular"[All Fields] AND "Atrophy"[All Fields] AND "Spinal"[All Fields]) OR "spinal muscular atrophy"[All Fields] OR ("Muscular"[All Fields] AND "Atrophy"[All Fields] AND "Spinal"[All Fields]) OR "muscular atrophy spinal"[All Fields]) AND "intermediate type"[Text Word]) OR (("muscular atrophy, spinal"[MeSH Terms] OR ("Muscular"[All Fields] AND "Atrophy"[All Fields] AND "Spinal"[All Fields]) OR "spinal muscular atrophy"[All Fields] OR ("Muscular"[All Fields] AND "Atrophy"[All Fields] AND "Spinal"[All Fields]) OR "muscular atrophy spinal"[All Fields]) AND "type iii"[Text Word]) OR "spinal muscular atrophy type iii"[Text Word] OR "spinal muscular atrophy infantile"[Text Word] OR "spinal muscular atrophy juvenile"[Text Word] OR "spinal muscular atrophy type 3"[Text Word] OR "spinal muscular atrophy type iii"[Text Word] OR "type iii spinal muscular atrophy"[Text Word] OR "werdnig hoffman disease"[Text Word] OR "werdnig hoffmann disease"[Text Word] OR "werdnig hoffmann disease"[Text Word]) AND ("survival of motor neuron 1 protein"[MeSH Terms] OR (("SMN"[All Fields] AND ("protein s"[All Fields] OR "proteinous"[All Fields] OR "proteins"[MeSH Terms] OR "proteins"[All Fields] OR "Protein"[All Fields])) AND "spinal muscular atrophy"[Text Word]) OR ((("mortality"[MeSH Subheading] OR "mortality"[All Fields] OR "survival"[All Fields] OR "survival"[MeSH Terms] OR "survivability"[All Fields] OR "survivable"[All Fields] OR "survivals"[All Fields] OR "survive"[All Fields] OR "survived"[All Fields] OR "survives"[All Fields] OR "surviving"[All Fields]) AND ("motor neurons"[MeSH Terms] OR ("Motor"[All Fields] AND "neurons"[All Fields]) OR "motor neurons"[All Fields] OR ("Motor"[All Fields] AND "neuron"[All Fields]) OR "motor neuron"[All Fields])) AND "protein 1"[Text Word])) AND ("Screening"[Text Word] OR "diagnostic screening programs"[MeSH Terms] OR "diagnostic screening program"[Text Word] OR (("Program"[All Fields] OR "program s"[All Fields] OR "programe"[All Fields] OR "programed"[All Fields] OR "programes"[All Fields] OR "programing"[All Fields] OR "programmability"[All Fields] OR "programmable"[All Fields] OR "programmably"[All Fields] OR "programme"[All Fields] OR "programme s"[All Fields] OR "programmed"[All Fields] OR "programmer"[All Fields] OR "programmer s"[All Fields] OR "programmers"[All Fields] OR "programmes"[All Fields] OR "programming"[All Fields] OR "programmings"[All Fields] OR "Programs"[All Fields]) AND "diagnostic screening"[Text Word]) OR (("Program"[All Fields] OR "program s"[All Fields] OR "programe"[All Fields] OR "programed"[All Fields] OR "programes"[All Fields] OR "programing"[All Fields] OR "programmability"[All Fields] OR "programmable"[All Fields] OR "programmably"[All Fields] OR "programme"[All Fields] OR "programme s"[All Fields] OR "programmed"[All Fields] OR "programmer"[All Fields] OR "programmer s"[All Fields] OR "programmers"[All Fields] OR "programmes"[All Fields] OR "programming"[All Fields] OR "programmings"[All Fields] OR "Programs"[All Fields]) AND "diagnostic screening"[Text Word]) OR "screening program diagnostic"[Text Word] OR "screening programs diagnostic"[Text Word])	

TOTAL = 593
Endnote 42 duplicates = 551
Rayyan 24 duplicates = 527 studies
